# Oakland score to identify low-risk patients with lower gastrointestinal bleeding performs well among emergency department patients

**DOI:** 10.1186/s12245-025-00815-5

**Published:** 2025-02-03

**Authors:** Daniel D. DiLena, Sean C. Bouvet, Madeline J. Somers, Maqdooda A. Merchant, Theodore R. Levin, Adina S. Rauchwerger, Dana R. Sax

**Affiliations:** 1 Division of Research, Kaiser Permanente Northern California, 4480 Hacienda Dr, Pleasanton, CA 94588 USA; 2 Department of Emergency Medicine, Kaiser Permanente San Francisco Medical Center, San Francisco, CA USA; 3 Department of Gastroenterology, Kaiser Permanente Antioch Medical Center, Antioch, CA USA; 4 Department of Emergency Medicine, Kaiser Permanente Oakland Medical Center, Oakland, CA USA

**Keywords:** Lower gastrointestinal bleeding, Risk stratification, Emergency medicine

## Abstract

**Background:**

The Oakland Score predicts risk of 30-day adverse events among hospitalized patients with lower gastrointestinal bleeding (LGIB) possibly identifying patients who may be safe for discharge. The Oakland Score has not been studied among emergency department (ED) patients with LGIB. The Oakland Score composite outcome includes re-bleeding, defined as additional blood transfusion requirements and/or a further decrease in hematocrit (Hct) >/= 20% after 24 h in clinical stability; red blood cell transfusion; therapeutic intervention to control bleeding, including surgery, mesenteric embolization, or endoscopic hemostasis; in-hospital death, all cause; and re-admission with further LGIB within 28 days. Prediction variables include age, sex, previous LGIB admission, systolic blood pressure, heart rate, and hemoglobin concentration, and scores range from 0 to 35 points, with higher scores indicating greater risk.

**Methods:**

Retrospective cohort study of adult (≥ 18 years old) patients with a primary ED diagnosis of LGIB across 21 EDs from March 1st, 2018, through March 1st, 2020. We excluded patients who were more likely to have upper gastrointestinal bleeding (esophago-gastroduodenoscopy without LGIB evaluation), patients who left against medical advice or prior to ED provider evaluation, ED patients without active health plan membership, and patients with incomplete Oakland Score variables. We assessed predictive accuracy by reporting the area under the receiver operator curve (AUROC) and sensitivity, specificity, positive and negative predictive values, and positive and negative likelihood ratios at multiple clinically relevant thresholds.

**Results:**

We identified 8,283 patients with LGIB, 52% were female, mean age was 68, 49% were non-White, and 27% had an adverse event. The AUROC for predicting an adverse event was 0.85 (95% CI 0.84–0.86). There were 1,358 patients with an Oakland Score of </=8; 4.9% had an adverse event, and sensitivity of the Oakland Score at this threshold was 97% (95% CI 96%−98%).

**Conclusion:**

The Oakland Score had high predictive accuracy among ED patients with LGIB. Prospective evaluation is needed to understand if the risk score could augment ED decision-making and improve outcomes and resource utilization.

**Supplementary Information:**

The online version contains supplementary material available at 10.1186/s12245-025-00815-5.

## Introduction

### Background

Lower gastrointestinal bleeding (LGIB), or bleeding distal to the ligament of Treitz [1], contributes to approximately 100,000 hospital admissions in the U.S. annually [2–4]. Compared to upper gastrointestinal bleeding (UGIB), LGIB is less likely to present with hemorrhagic shock or require red blood cell (RBC) transfusions, and the in-hospital mortality rate for LGIB is lower, approximately 2–4% [5–7]. It is estimated that in the U.S. alone, healthcare for gastrointestinal (GI) bleeding incurred direct costs of $5 billion in 2014 [8], with individual costs for LGIB between $22,142 and $28,749 per hospitalization, or $4,492 per bed-day [9]. 

Although bleeding often resolves spontaneously, elderly patients and those with co-morbid conditions face higher risks of adverse outcomes [5, 7, 10]. Some data suggest that rates of hospitalization, resource utilization, and length of hospital stay for LGIB have surpassed UGIB [11–13]. Because most cases are self-limited, there is an increased interest in developing prediction models to identify patients that may be safely managed outside the hospital.

### Importance

While validated risk scores exist for UGIB, decision support for LGIB is needed. The 2023 American College of Gastroenterology (ACG) guideline recommended using risk stratification tools such as the Oakland Score to triage hospitalized patients [2]. The Oakland Score predicts likelihood of safe hospital discharge, defined as the absence of all of the following after presentation: re-bleeding, defined as additional blood transfusion requirements and/or a further decrease in hematocrit (Hct) >/= 20% after 24 h in clinical stability; RBC transfusion; therapeutic intervention to control bleeding, including surgery, mesenteric embolization, or endoscopic hemostasis; in-hospital death, all cause; and re-admission with further LGIB within 28 days [14]. The original Oakland Score, derived and validated by Oakland et al. in 2017 among a nationally representative sample of hospitalized patients with LGIB in the U.K., used seven variables, including age, sex, history of LGIB, rectal examination findings, heart rate, systolic blood pressure, and hemoglobin level, to provide clinicians with a risk score between 0 and 35. Lower scores suggest patients at lower risk for adverse outcomes. The subsequent external validation study, which dropped the digital rectal examination (DRE) finding variable from the score due to limited electronic availability and included over 46,000 patients from 14 hospitals across the U.S., found that a threshold of ≤ 8 or ≤ 10 identified patients at very low risk for an adverse event, with sensitivity of 98.4% and 96.0%, respectively [15]. 

### Goals

Prior validation studies of the Oakland Score have been limited to hospitalized patients [11, 15]. A recent single center Korean study of 376 patients found the Oakland Score was highly sensitive among low-risk ED patients [16]. Given the majority of unscheduled hospital admissions come through the ED [17], our goal was to assess how the Oakland Score performs in an ED population prior to the decision to admit to the hospital. We describe patient characteristics and outcomes among a large cohort of ED patients with LGIB and report performance of the Oakland Score to identify low-risk patients who may be safe for outpatient management.

## Methods

### Study design and setting

This retrospective cohort study was conducted among health plan members of Kaiser Permanente Northern California (KPNC) across 21 medical centers with associated EDs. KPNC is a large, integrated healthcare delivery system providing care for over 4.5 million members, including over 1.5 million annual ED visits. Of the 21 EDs, 13 had an observation unit during the study period, although protocols, number of beds, and primary treatment team (ED versus hospitalist) varied between sites. KPNC demographics reflect the ethnic and socioeconomic diversity of the surrounding areas [18]. KPNC utilizes an integrated and comprehensive electronic health record (EHR), including all ED, inpatient, and outpatient records with associated laboratory, pharmacy, and imaging data. The KPNC Institutional Review Board approved the study protocol and waived informed consent.

### Selection of participants

We identified adult (>/= 18 years) health plan members with an ED encounter for LGIB between March 1st, 2018, and March 1st, 2020, using *International Classification of Disease 10* (ICD-10 codes) to identify encounters with a primary diagnosis of LGIB or undifferentiated GI hemorrhage (Appendix, Table 1) [19]. To limit misclassification of patients with UGIB, we excluded patient encounters with a completed esophagogastroduodenoscopy (EGD) study without a concomitant study to evaluate for LGIB (specifically, we searched for evidence of colonoscopy, separating therapeutic from diagnostic, sigmoidoscopy, CT angiography, CT colonography, or tagged RBC scan) during hospitalization (Appendix, Table 2). We excluded patients who left against medical advice, eloped prior to ED provider evaluation, or who had incomplete or missing Oakland Score components [14]. Lastly, we excluded patients who had a non-KPNC ED encounter, for which full encounter data was not available, within seven days of the index ED visit, to ensure accurate capture of LGIB evaluation and outcomes.

**Table 1:** Oakland Score components with assigned points

Variable	Score Component Value
**Age group**	
</=39	0
40–69	1
>/=70	2
**Sex**	
Female	0
Male	1
**Previous hospitalization for LGIB**	
No	0
Yes	1
**Initial Heart Rate**,** beats/min**	
< \=69	0
70–89	1
90–109	2
>/=110	3
**Initial systolic blood pressure**,** mm Hg**	
50–89	5
90–110	4
120–129	3
130–159	2
>/=160	0
**Initial Hemoglobin concentration**,** g/dL**	
3.6–6.9	22
7.0–8.9	17
9.0–10.9	13
11.0–12.9	8
13.0–15.9	4
>/=16.0	0

Abbreviations: LGIB, Lower gastrointestinal bleeding

**Table 2:** Patient characteristics, overall study cohort and among those with and without an adverse event

	Overall, *N* = 8,283	Adverse Event, *n* = 2,243	No Adverse Event, *n* = 6,040	*p*-value^1^
**Age**,** mean (SD)**	67.7 (18.0)	73.4 (14.1)	65.6 (18.9)	< 0.001
**Male**,** n (%)**	4,022 (48.6)	1,145 (51.1)	2,877 (47.6)	0.005
**Race category**,** n (%)**				0.004
Asian	1,345 (16.2)	386 (17.2)	959 (15.9)	
Black	819 (9.9)	215 (9.6)	604 (10.0)	
Hispanic	1,433 (17.3)	340 (15.2)	1,093 (18.1)	
Other*	455 (5.5)	109 (4.9)	346 (5.7)	
Non-Hispanic White	4,231 (51.1)	1,193 (53.2)	3,038 (50.3)	
**Neighborhood deprivation index** ^**#**^	-0.2 (0.9)	-0.3 (0.8)	-0.2 (0.9)	0.09
**Arrival by ambulance**,** n (%)**	1,588 (19.2)	711 (31.7)	877 (14.5)	< 0.001
**Colonoscopy in previous 2 years**,** n (%)**	2,298 (27.7)	731 (32.6)	1,567 (25.9)	< 0.001
**LGIB hospitalization in previous 2 years n (%)**	633 (7.6)	281 (12.5)	352 (5.8)	< 0.001
**Co-morbid illnesses**,** n (%)^**				
Chronic Heart Failure	1,528 (18.4)	655 (29.2)	873 (14.5)	< 0.001
Liver disease	1,253 (15.1)	405 (18.1)	848 (14.0)	< 0.001
Renal failure	2,263 (27.3)	881 (39.3)	1,382 (22.9)	< 0.001
Coagulopathy	989 (11.9)	438 (19.5)	551 (9.1)	< 0.001
Total Elixhauser co-morbidity score	4.9 (3.5)	6.66 (3.6)	4.30 (3.3)	< 0.001
**Active medications**,** n (%)**				
Oral antiplatelets	3,630 (43.8)	1,369 (61.0)	2,261 (37.4)	< 0.001
Oral anticoagulants	2,412 (29.1)	884 (39.4)	1,528 (25.3)	< 0.001
**ED vital signs**,** mean (SD)**				
Heart rate, beats/min	84.1 (17.6)	87.4 (19.1)	82.9 (16.8)	< 0.001
Systolic blood pressure, mm Hg	135.9 (24.6)	125.8 (25.2)	139.6 (23.2)	< 0.001
**ED Laboratory values**,** mean (SD)**				
Hemoglobin, g/dL	11.6 (2.8)	9.0 (2.7)	12.6 (2.1)	< 0.001
Platelet count, 10^3/uL	241.5 (92.5)	247.4 (111.1)	239.3 (84.4)	0.6
White Blood Cell count /uL	8.8 (6.3)	9.4 (8.6)	8.6 (5.1)	< 0.001
Creatinine level mg/dL	1.2 (1.1)	1.5 (1.4)	1.1 (1.0)	< 0.001
INR	1.5 (1.1)	1.7 (1.4)	1.4 (0.9)	< 0.001
**ED Disposition**,** n (%)**				< 0.001
Hospital admission	2,289 (27.6)	1,362 (60.7)	927 (15.3)	
Observation	2,367 (28.6)	679 (30.3)	1,688 (27.9)	
ED discharge	3,509 (42.4)	161 (7.2)	3,348 (55.4)	
Transfer to another hospital	118 (1.4)	41 (1.8)	77 (1.3)	

Notes: Abbreviations: LGIB, Lower gastrointestinal bleeding; ED, emergency department; SD, standard deviation; INR, International normalized ratio

^1^ Wilcoxon rank sum test; Pearson’s Chi-squared test

*Other race includes American Indian or Alaska Native, Native Hawaiian or other Pacific Islander, and more than 1 race

^#^ The Neighborhood Deprivation Index for each census tract is based on 13 socioeconomic measures. Scores range from − 3.6 to 2.8, with higher values indicating more neighborhood deprivation (lower socioeconomic status)

^Co-morbid illness categories were taken from the Elixhauser Co-morbidity Index [19].

Anti-platelet medications included: Aspirin, also called acetylsalicylic acid (Aspirin, Asaphen, Entrophen, Novasen), Clopidogrel (Plavix), Prasugrel (Effient), Ticagrelor (Brilinta), Ticlopidine (Ticlid), Dipyridamole (Persantine), Cilostazol (Pletal), aspirin/dipyridmole (Aggrenox), Cangrelor, Vorapaxar, abciximab, caplacizumab, eptifibatide, tirofiban

Anti-coagulant medications included: Warfarin (Coumadin), Apixaban (Eliquis), edoxaban (Savaysa), Fondaparinus (Arixtra), Enoxaparin (Lovenox), argatroban (Acova), bivalirudin (Angiomax), dabigatran (Pradaxa), desirudin (Iprivask or Hirudin), ardeparin, betrixaban, dalteparin, danaparoid

Variables with missingness: Platelet count (*n* = 102 missing), Creatinine (*n* = 184 missing), INR (*n* = 2492 missing, of which 2002 were among patients without an adverse event)

### Variables

We extracted the six Oakland Score variables (dropping DRE findings because of inconsistent electronic capture) from the EHR, shown in Table 1 with their assigned points. Each eligible patient encounter was assigned a score between 0 and 35, with hemoglobin carrying the most weight (up to + 22 points). We used the first ED heart rate, systolic blood pressure, and hemoglobin concentration to calculate the score.

We collected patient demographics, healthcare utilization, and medications (anti-platelets and anti-coagulants) from the EHR and vital signs and laboratory values from the ED visit. We report patient co-morbidity burden using the Elixhauser Co-morbidity Index, both total Elixhauser score and key diagnostic categories included within the index [20]. We assessed neighborhood socioeconomic status at the census block track level, using the most recent American Community Service values that are built on 2010 Census data [21]. We assessed race and ethnicity data (Asian, Black, Hispanic, non-Hispanic White, other [including American Indian or Alaska Native, Native Hawaiian or other Pacific Islander, and multiple races or ethnicities], unknown, or missing).

### Outcome

The primary outcome was the composite outcome used in the derivation and validation studies [14, 15], ‘safe for hospital discharge,’ as previously defined (Appendix, Table 2). Re-bleeding among hospitalized patients was defined as 20% or greater decrease in hematocrit after 24 h in which the hematocrit was stable (< 10% variation). We defined re-bleeding among discharged patients as a repeat ED visit for LGIB within 72 h or a repeat outpatient hematocrit within seven days of discharge that was 20% less than the last ED hematocrit.

### Statistical analysis

We describe patient characteristics among the full sample and among those with and without an adverse event, shown in Table 2. We report rates of the composite outcome score for each adverse event at multiple Oakland Score thresholds. We assessed predictive accuracy using area under the receiver operator curve (AUROC) and precision recall curve from a logistic regression model. We examined sensitivity, specificity, positive predictive value (PPV), negative predictive value (NPV), likelihood ratios, and F1 scores for several cutoffs of the Oakland Score (score </=7, </=8, </=9, </=10, </=15). We performed a variable importance list using a logistic regression model with all Oakland Score variables. Finally, as a sensitivity analysis, we examined the utility of additional variables (anti-platelet or anticoagulant medications and colonoscopy in the past two years) in the performance of a logistic regression with the validated Oakland Score predictors.

There was no missingness in the components of the Oakland Score (cases with missing variables were excluded as with the original studies) or among the outcome measures.

Data management and cleaning was done in SAS version 9.4 (SAS Institute Inc), and statistical analysis was performed using R version 4.3.1. Two-sided α < 0.05 indicated statistical significance.

## Results

**Fig. 1:**
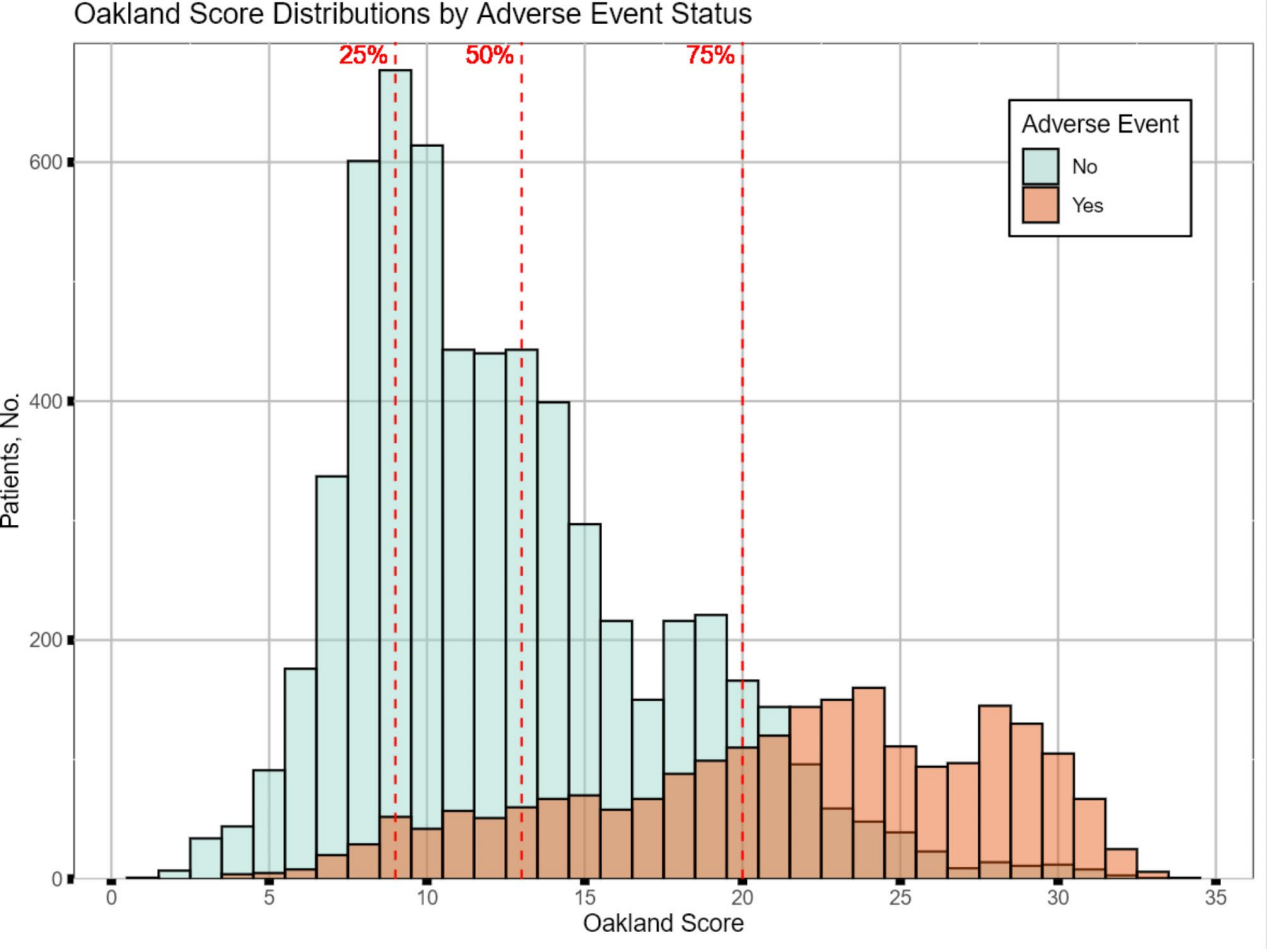
Oakland Score distribution by adverse event status

There were 8,283 patients with an ED visit for LGIB during the study period who met study inclusion criteria (Appendix, Fig. 1). The median age was 67.7 years, 48.6% were male, and 48.9% were non-White (Table 2). We found 3,627 (43.8%) were directly discharged from the ED or transferred, and an additional 2,367 (28.6%) were discharged after a brief observation stay (generally < 24 h); the remaining 2,289 (27.6%) were admitted to the hospital. There were high rates of co-morbid illness, including liver, kidney, and heart disease, and 43.8% and 29.1% of patients were taking anti-platelet medications and anti-coagulant medications, respectively.

Overall, 2,243 patients (27.1%) experienced an adverse event. Patients without an adverse event were younger (mean age 65.6 versus 73.4) with lower rates of prior admissions for LGIB (5.8% versus 12.5%), lower rates of kidney, liver, heart disease, or coagulopathy, and they were less likely to be taking oral anticoagulant or antiplatelet medications and had higher presenting hemoglobin values (12.6 g/dL versus 9.0 g/dL).

The median Oakland Score was 13 with an interquartile range of 9 to 20. Figure 1 shows the overlap of Oakland Scores with rates of adverse events, demonstrating that increasing scores were associated with higher probability of adverse events. We found 1,358 (16.4%) had an Oakland Score of </=8 and 2,743 (33.1%) had a score of </=10. Rates of the composite Oakland Score outcome and rates of individual adverse events at different low-risk thresholds are seen in Table 3. We found 4.9% and 5.9% of patients had an adverse event using a threshold of </=8 or </=10, respectively, with red blood cell transfusion being the most common adverse event. Transfusion also contributed to nearly all of the increase in adverse events moving from a score of </=8 to </=10. There was no increase in the proportion of patients who died or required surgery, a 0.1% increase in the proportion that required mesenteric embolization, and a 0.1% decrease in the proportion that required a therapeutic colonoscopy.

**Table 3:** Rates of the Oakland Score composite outcome as well as individual adverse events at various lower risk thresholds

Oakland Score threshold	</=7	</=8	</=9	</=10
n (%)	728 (8.8)	1,358 (16.4)	2,087 (25.2)	2,743 (33.1)
Any adverse event (%)	38 (5.2)	67 (4.9)	119 (5.7)	161 (5.9)
Mesenteric embolization (%)	1 (0.1)	3 (0.2)	5 (0.2)	7 (0.3)
Therapeutic colonoscopy (%)	11 (1.5)	24 (1.8)	37 (1.8)	48 (1.7)
Surgery (%)	1 (0.1)	1 (< 0.1)	1 (< 0.1)	1 (< 0.1)
RBC transfusion (%)	16 (2.2)	27 (2.0)	56 (2.7)	80 (2.9)
Re-bleeding among ED discharged patients				
Repeat ED visit with hematocrit drop (%)	4 (0.5)	5 (0.4)	10 (0.5)	15 (0.5)
Hematocrit drop within 7 days based on outpatient laboratory examination (%)	8 (1.1)	10 (0.7)	16 (0.8)	23 (0.8)
Re-bleeding among hospitalized patients (%)	0 (0.0)	0 (0.0)	0 (0.0)	2 (< 0.1)
28-day hospital readmission for LGIB (%)	2 (0.3)	3 (0.2)	8 (0.4)	10 (0.4)
Hospital death (%)	2 (0.3)	2 (0.1)	3 (0.1)	3 (0.1)

Notes: Abbreviations: RBC, red blood cell; LGIB, lower gastrointestinal bleed; ED, emergency department

The Oakland Score maintained a high sensitivity (97.0%) for safe discharge among patients scoring 8, with a specificity of 21.4%. Increasing the threshold to 10 points lowered the sensitivity to 92.8% and increased specificity to 42.8%. Additional Oakland Score performance characteristics at various thresholds can be seen in Table 4.

**Table 4:** Performance characteristics of the Oakland Score at various thresholds with 95% confidence intervals

Oakland Score Cutoff	*n*	Sensitivity	Specificity	PPV	NPV	Positive LR	Negative LR
7	728	98% (98–99%)	11% (11–12%)	29% (28–30%)	95% (93–96%)	1.11 (1.1–1.12)	0.15 (0.11–0.2)
8	1358	97% (96–98%)	21% (20–22%)	31% (30–33%)	95 (94–96%)	1.23 (1.22–1.25)	0.14 (0.11–0.18)
9	2087	95% (94–96%)	33% (31–34%)	34% (33–35%)	94% (93–95%)	1.40 (1.38–1.43)	0.16 (0.14–0.19)
10	2743	93% (92–94%)	43% (41–44%)	38% (36–39%)	94% (93–95%)	1.62 (1.58–1.66)	0.17 (0.14–0.2)
15	5070	79% (77–81%)	76% (75–77%)	55% (54–57%)	91% (90–92%)	3.33 (3.17–3.5)	0.27 (0.25–0.3)

Notes: Abbreviations: PPV, positive predictive value; NPV, negative predictive value; Positive LR, likelihood ratio

**Fig. 2:**
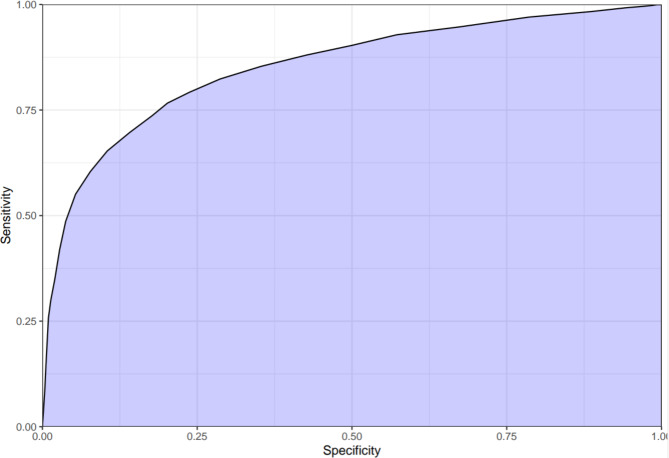
Area under the receiver operator curve for the Oakland Score

**Fig. 3:**
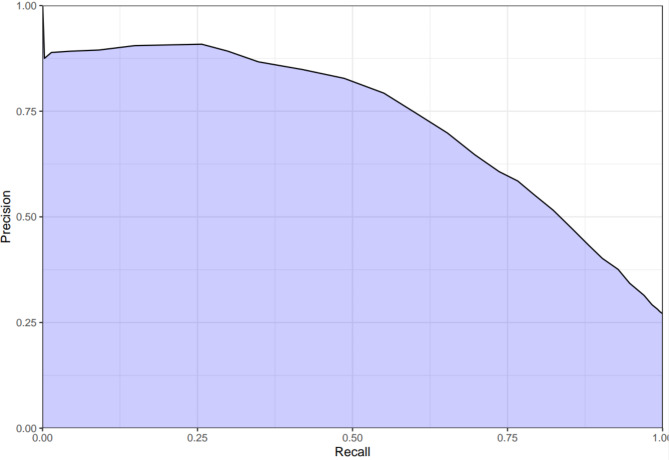
Area under the precision recall curve for the Oakland Score

Figures 2 and 3 show the AUROC and area under the precision recall curve (AUPRC), respectively. The AUROC for predicting a patient safe for discharge was 0.85 (95% CI, 0.84–0.86), while the AUPRC was 0.85. The most important variables in predicting an adverse event, in order of influence on the outcome, were hemoglobin concentration, systolic blood pressure, age >/=70, and HR >/=110 (Appendix, Fig. 2). The variables with limited influence on the outcome included use of anti-coagulant medications, heart rate of 70–89, history of LGIB in the prior two years, male gender, and history of colonoscopy in the prior two years.

We found that overall, ED admission decision-making correlated with predicted risk of an adverse event, with ED discharge more common among lower-risk patients and admission of higher-risk patients. Table 5 shows that 8.4% of very low-risk patients (score </=7) and 8.0% of low-risk patients (score 8–9) were admitted to the hospital, while 19.0% of moderate-risk (score 11–14) and 18.8% of high-risk patients (score >/=15) were discharged, and adverse event rates were 12.0% and 51.7%, respectively, in these risk strata. Among discharged patients who were moderate risk, 22 (2.3%) had a re-bleed, and five (0.5%) had LGIB readmission. Among discharged patients who were high risk, 33 (5.9%) had a re-bleed, and seven (1.3%) had LGIB readmission. The ED discharge rate of high-risk patients varied nearly 3-fold across the 21 EDs in our health system.

**Table 5:** ED disposition (direct discharge, treatment in an observation area followed by discharge, or hospital admission), adverse event rates, and ED mortality by predicted risk (Oakland Score threshold)

Risk category (Oakland Score threshold)	Adverse event n (%)	*n* (% ) within risk strata	Discharged *n* (%)	Observed *n* (%)	Admitted *n* (%)	Died in ED *n* (%)
Very low ( < = 7)	37 (5.1)	728 (8.8)	537 (73.8)	130 (17.9)	61 (8.4)	0 (0.0)
Low (Score 8–9)	83 (6.1)	1359 (16.4)	986 (72.6)	264 (19.4)	109 (8.0)	0 (0.0)
Low/Moderate (Score = 10)	43 (6.6)	656 (7.9)	389 (59.3)	168 (25.6)	99 (15.1)	0 (0.0)
Moderate (Score 11–14)	235 (12.0)	1960 (23.7)	916 (46.7)	669 (34.1)	375 (19.1)	0 (0.0)
High (Score > = 15)	1850 (51.7)	3580 (43.2)	672 (18.8)	1135 (31.7)	1763 (49.3)	10 (0.3)

## Discussion

To our knowledge, this is the first large, community-based, multi-center external validation of the Oakland Score to assess whether the prediction tool can accurately identify ED patients with LGIB who are at low risk for an adverse event and therefore may be appropriate for safe ED outpatient care. Among a diverse cohort of 8,283 patients across 21 EDs, we found the Oakland Score was highly sensitive to identifying low-risk patients. Generally, ED provider admission decisions correlated with predicted risk, although we did find wide variation between EDs regarding discharge of moderate and high-risk patients, and some discharged patients experienced adverse events, suggesting clinical decision support might help aid risk stratification.

Because we shifted the study population further upstream (from hospitalized patients to ED patients), it included a larger proportion of low and low/moderate risk patients compared to the Oakland Score derivation and validation studies, which included only hospitalized patients. ED patients include those across the risk spectrum, including younger, healthier patients who are less likely to have a severe cause of LGIB or experience an adverse event. Only 27.1% of patients in our cohort experienced an adverse event, compared to 52.1% among the hospitalized cohort in the external validation study [15]. We found 16.4% of patients were predicted to be low risk (Oakland Score of </=8) in our population, while only 8.7% were predicted to be low risk among hospitalized patients in the external Oakland Score validation study [15]. 

Our findings of risk model performance suggest the Oakland Score can be used to safely identify low-risk patients who may be safe for discharge. Using a </=8 threshold, the Oakland Score was 97% sensitive to identify patients who had an adverse event, comparable to the sensitivity found in the external validation study among hospitalized patients (98.4%) [15]. The recent Korean validation study of the Oakland Score among ED patients found a sensitivity of 100% using a </=8 threshold.

Several risk scores have been proposed to help risk stratify patients with LGIB, including the Strate, NOBLADS, BLEED, SHA_2_PE, and Oakland scores, each with slightly different goals and predictive accuracy [2, 22, 23]. The Strate score uses seven clinical variables, does not require bloodwork, and is designed to predict severe LGIB. The NOBLADS score consists of eight variables and is designed to predict severe bleeding. The BLEED score consists of five variables and is designed to predict an in-hospital complication. The SHA_2_PE score consists of seven variables and was developed among ED patients with LGIB. In an external validation study of the SHA_2_PE score and comparison to the Oakland Score among a cohort of 595 hospitalized patients with LGIB, both scores performed well; the AUCs for the Oakland Score and SHA_2_PE score were 0.85 (95% CI 0.82–0.89) and 0.797 (95% CI 0.75–0.84), respectively [23, 24]. 

We found the Oakland Score had an AUROC of 0.85 (95% CI 0.84–0.86) suggesting good discriminative performance among ED patients with LGIB. Similar to findings in the validation study of hospitalized patients [15], specificity and positive predictive values were low among patients predicted to be low risk, suggesting the risk estimates are safe and conservative. This matches providers’ desire for a decision support tool that limits misclassifying high-risk patients as low risk.

We found 42.4% of patients were directly discharged from the ED, and an additional 28.6% were discharged after a brief observation stay. In general, ED providers discharged patients predicted to be lower risk, and adverse events among discharged patients were infrequent. We found that 8.1% of low-risk patients were hospitalized, and 18.8% of high-risk patients were discharged, suggesting an opportunity to better match predicted risk with intensity of care (hospitalization) with a risk stratification tool. However, it is important to recognize that a variety of factors, many of which are not captured by the Oakland Score, may influence a provider’s decision to admit a patient, including medical comorbidities, social determinants of health, and other clinical considerations.

Hospital admissions for LGIB are costly [9], and prior studies show that bleeding is self-limited in most patients [3, 5]. Utilizing risk prediction tools like the Oakland Score during admission decision-making could help optimize resource allocation while also mitigating potential negative effects of prolonged or unnecessary hospital stays [2]. Furthermore, the Oakland Score may assist providers in identifying true high-risk patients who may benefit from close observation and treatment in the hospital. ACG guidelines on the management of LGIB support use of risk stratification tools including the Oakland Score and threshold of </=8, and our findings among ED patients suggest this score may be useful to stratify patients prior to the decision to admit or discharge a patient. Other scores described in the ACG guidelines include the NOBLADS score and the SHA_2_PE score for identifying low-risk patients [2]. Risk prediction scores are designed to support clinical decision making and should not be used in lieu of a clinician’s judgment.

Oakland et al. suggest that increasing the threshold from 8 to 10 points identifies a larger portion of low-risk patients while maintaining good discriminative performance. Identifying the ideal threshold score for safe discharge requires balancing the risk of higher rates of adverse events among patients identified as low risk and safe for discharge with the goal of optimizing resource utilization decisions. Increasing the “low-risk” threshold from </=8 to </=10 doubled the number of patients in this category (from 16.4 to 33.1%), while sensitivity decreased from 97 to 93%, and rates of adverse events increased from 4.9 to 5.9%. This 1% difference in adverse events was predominantly driven by need for transfusion and not by death or invasive procedures.

More than two-thirds of patients in our cohort experienced no adverse outcomes as previously defined. The most common adverse event was RBC transfusion, consistent with other large observational studies of patients with LGIB [4, 15]. The Oakland Score composite outcome includes a broad spectrum of invasive interventions and serious outcomes. Among the 2,743 patients identified as low risk using the </=10 threshold, rates of serious invasive procedures or in-hospital mortality were low: less than 10 patients required mesenteric embolization or surgery and three patients died. There were 38 patients who had evidence of a re-bleed after ED discharge in this group, although only 10 were admitted to the hospital for LGIB within 28 days.

As with the prior external validation study [15], we found the score maintained high predictive accuracy even without DRE findings. The remaining six variables were sufficient to maintain high sensitivity to identify low-risk patients. Among these, hemoglobin concentration and systolic blood pressure had the highest clinical significance in predicting an adverse event, aligning with their respective weightings in the score calculation. Although we hypothesized that incorporating additional clinical variables (such as use of anti-platelets or anti-coagulants) might further improve model discrimination, these variables were found to be non-predictive in the Oakland Score derivation study [14], and had little influence on the outcome in our dataset (Appendix, Fig. 2). A simple model, including only demographic factors, vital signs, and one blood test, allows for ease of calculation and may increase interpretability and provider trust, compared to more complex models.

Further study is needed to prospectively validate the Oakland Score at the point of care and compare ED provider gestalt with risk predictions. Additional investigations should also include model performance among different populations to determine potential for bias.

### Limitations

The main limitation of this study is its retrospective design. Origin of bleeding is difficult to confirm retrospectively among patients with an ED diagnosis code of undifferentiated GI hemorrhage. Although we tried to limit inclusion of patients with UGIB, there may have been some mis-categorization. There may be unmeasured severity of illness or patient preferences that may have driven hospital admission decision-making.

Additionally, other systems may have less access to follow up care after ED discharge, and this may impact risk thresholds and admission decision-making, potentially limiting the generalizability of our findings.

## Conclusion

We present findings from a large, diverse, multi-center external validation study of the Oakland Risk Score among ED patients diagnosed with LGIB. We found that the Oakland Score maintained high predictive accuracy in identifying low-risk patients who may be amenable to outpatient care in this novel application of the score to patients prior to the hospital admission decision. Next steps could include a prospective implementation study to ensure the safety of risk estimates.

## Electronic supplementary material

Below is the link to the electronic supplementary material.

Supplementary Material 1

## Data Availability

The datasets used and/or analyzed during the current study are available from the corresponding author on reasonable request.

## References

[1] Zheng NS, Tsay C, Laine L, Shung DL. Trends in characteristics, management, and outcomes of patients presenting with gastrointestinal bleeding to emergency departments in the United States from 2006 to 2019. Aliment Pharmacol Ther. 2022;56(11–12):1543–55. 10.1111/apt.17238. 36173090 10.1111/apt.17238 PMC9669230

[2] Sengupta N, Feuerstein JD, Jairath V, et al. Management of patients with Acute Lower gastrointestinal bleeding: an updated ACG Guideline. Am J Gastroenterol. 2023;118(2):208–31. 10.14309/ajg.0000000000002130. 36735555 10.14309/ajg.0000000000002130

[3] Hreinsson JP, Gumundsson S, Kalaitzakis E, Björnsson ES. Lower gastrointestinal bleeding: incidence, etiology, and outcomes in a population-based setting. Eur J Gastroenterol Hepatol. 2013;25(1):37–43. 10.1097/MEG.0b013e32835948e3. 23013623 10.1097/MEG.0b013e32835948e3

[4] Lanas A, García-Rodríguez LA, Polo-Tomás M, et al. Time trends and impact of upper and lower gastrointestinal bleeding and perforation in clinical practice. Am J Gastroenterol. 2009;104(7):1633–41. 10.1038/ajg.2009.164. 19574968 10.1038/ajg.2009.164

[5] Oakland K, Guy R, Uberoi R, et al. Acute lower GI bleeding in the UK: patient characteristics, interventions and outcomes in the first nationwide audit. Gut. 2018;67(4):654–62. 10.1136/gutjnl-2016-313428. 28148540 10.1136/gutjnl-2016-313428

[6] Adegboyega T, Rivadeneira D, Lower GI, Bleeding. An update on incidences and causes. Clin Colon Rectal Surg. 2020;33(1):28–34. 10.1055/s-0039-1695035. 10.1055/s-0039-1695035 PMC6946606 31915423

[7] Marion Y, Lebreton G, Le Pennec V, Hourna E, Viennot S, Alves A. The management of lower gastrointestinal bleeding. J Visc Surg. 2014;151(3):191–201. 10.1016/j.jviscsurg.2014.03.008. 24768401 10.1016/j.jviscsurg.2014.03.008

[8] Peery AF, Crockett SD, Murphy CC, et al. Burden and cost of gastrointestinal, liver, and pancreatic diseases in the United States: Update 2021. Gastroenterology. 2022;162(2):621–44. 10.1053/j.gastro.2021.10.017. 34678215 10.1053/j.gastro.2021.10.017 PMC10756322

[9] Navaneethan U, Njei B, Venkatesh PGK, Sanaka MR. Timing of colonoscopy and outcomes in patients with lower GI bleeding: a nationwide population-based study. Gastrointest Endosc. 2014;79(2):297–e30612. 10.1016/j.gie.2013.08.001. 24060518 10.1016/j.gie.2013.08.001

[10] Camus M, Jensen DM, Ohning GV, et al. Comparison of three risk scores to predict outcomes of severe lower gastrointestinal bleeding. J Clin Gastroenterol. 2016;50(1):52–8. 10.1097/MCG.0000000000000286. 25599218 10.1097/MCG.0000000000000286 PMC4504830

[11] Fong HY. External validation of the Oakland score to assess safe hospital discharge among adult patients with acute lower gastrointestinal bleeding in an accident and emergency department in Hong Kong. Hong Kong J Emerg Med Published Online May. 2023;19:10249079231175434. 10.1177/10249079231175434.

[12] Oakland K, Chadwick G, East JE, et al. Diagnosis and management of acute lower gastrointestinal bleeding: guidelines from the British Society of Gastroenterology. Gut. 2019;68(5):776–89. 10.1136/gutjnl-2018-317807. 30792244 10.1136/gutjnl-2018-317807

[13] Vora P, Pietila A, Peltonen M, Brobert G, Salomaa V. Thirty-year incidence and mortality trends in Upper and Lower gastrointestinal bleeding in Finland. JAMA Netw Open. 2020;3(10):e2020172. 10.1001/jamanetworkopen.2020.20172. 33034641 10.1001/jamanetworkopen.2020.20172 PMC7547368

[14] Oakland K, Jairath V, Uberoi R, et al. Derivation and validation of a novel risk score for safe discharge after acute lower gastrointestinal bleeding: a modelling study. Lancet Gastroenterol Hepatol. 2017;2(9):635–43. 10.1016/S2468-1253(17)30150-4. 28651935 10.1016/S2468-1253(17)30150-4

[15] Oakland K, Kothiwale S, Forehand T, et al. External validation of the Oakland Score to assess Safe Hospital Discharge among adult patients with Acute Lower gastrointestinal bleeding in the US. JAMA Netw Open. 2020;3(7):e209630. 10.1001/jamanetworkopen.2020.9630. 32633766 10.1001/jamanetworkopen.2020.9630 PMC7341175

[16] Whiteway J, Yim S, Leong N, Shah A. External validation of the Oakland Score for Predicting Safe Discharge in patients presenting with lower gastrointestinal bleeding at the William Harvey Hospital in the United Kingdom. Cureus. 2024;16(3):e55497. 10.7759/cureus.55497. 38440205 10.7759/cureus.55497 PMC10911392

[17] Kocher KE, Dimick JB, Nallamothu BK. Changes in the source of unscheduled hospitalizations in the United States. Med Care. 2013;51(8):689–98. 10.1097/MLR.0b013e3182992c7b. 23752257 10.1097/MLR.0b013e3182992c7b

[18] Davis AC, Voelkel JL, Remmers CL, Adams JL, McGlynn EA. Comparing Kaiser Permanente Members to the General Population: implications for generalizability of Research. Perm J. 2023;27(2):87–98. 10.7812/TPP/22.172. 37170584 10.7812/TPP/22.172 PMC10266863

[19] ICD-10: international statistical classification of diseases and related health problems: tenth revision. World Health Organization; 2004. Accessed April 9, 2024. https://iris.who.int/handle/10665/42980

[20] Elixhauser A, Steiner C, Harris DR, Coffey RM. Comorbidity measures for Use with Administrative Data. Med Care. 1998;36(1):8–27. 9431328 10.1097/00005650-199801000-00004

[21] Messer LC, Laraia BA, Kaufman JS, et al. The development of a standardized neighborhood deprivation index. J Urban Health. 2006;83(6):1041–62. 10.1007/s11524-006-9094-x. 17031568 10.1007/s11524-006-9094-x PMC3261293

[22] Almaghrabi M, Gandhi M, Guizzetti L, et al. Comparison of risk scores for lower gastrointestinal bleeding: a systematic review and Meta-analysis. JAMA Netw Open. 2022;5(5):e2214253. 10.1001/jamanetworkopen.2022.14253. 35622365 10.1001/jamanetworkopen.2022.14253 PMC9142877

[23] Gonzalez-Gonzalez L, Iborra I, Fortuny M, et al. External validation of the SHA2PE score and its comparison to the Oakland score for the prediction of safe discharge in patients with lower gastrointestinal bleeding. Surg Endosc. 2024;38(8):4468–75. 10.1007/s00464-024-10953-1. 38902406 10.1007/s00464-024-10953-1

[24] Aoki T, Yamada A, Nagata N, Niikura R, Hirata Y, Koike K. External validation of the NOBLADS score, a risk scoring system for severe acute lower gastrointestinal bleeding. PLoS ONE. 2018;13(4):e0196514. 10.1371/journal.pone.0196514. 29698506 10.1371/journal.pone.0196514 PMC5919702

