# Supplementary material for: Oakland score to identify low-risk patients with lower gastrointestinal bleeding performs well among emergency department patients
Source: Int J Emerg Med. 2025 Feb 3;18:19. doi: 10.1186/s12245-025-00815-5 (PMC11792187; doi:10.1186/s12245-025-00815-5)
Supplement: Supplementary file 1 — Supplementary Material 1 [file 12245_2025_815_MOESM1_ESM.docx]

Appendix

Table 1: ICD-10 codes used for Study Cohort inclusion

| **Diagnostic category** | **ICD 10 codes** |
| --- | --- |
| Lower Gastrointestinal bleeding | K57.01, K57.11, K57.13, K57.21, K57.31, K57.33, K57.41, K57.51, K57.53, K57.81, K57.91, K57.93 |
| Melena | K92.1 |
| Hematochezia | K64.0, K64.1, K64.2, K64.3, K64.8 |
| Gastrointestinal hemorrhage | K55.21, K62.5, K92.2 |

Table 2: Procedure Codes

| **Procedure** | **Code type** | **Code** |
| --- | --- | --- |
| Esophagogastroduodenoscopy (EGD) | ICD-9 | 43244B, 43247, 189464, 202724, 204458, 204777, 206395, 207482 |
|  | ICD-10 | 099700Z, 0BB13ZX, 0BNK0ZZ, 0CNV8ZZ, 0CRW0J0, 0CRXXJ0, 0CRXXJ1, 0CSWXZ0, 0DCC0ZZ, 0DCE4ZZ, 0DCE7ZZ, 0DCE8ZZ, 0DCF0ZZ, 0DCF8ZZ, 0DCL0ZZ, 0DCM7ZZ, 0DCW3ZZ, 0DCW4ZZ, 0DV37DZ, 0GB00ZX, 0GB04ZZ, 0GB10ZX, 0GB10ZZ, 0GB13ZX, 0GTH0ZZ, 0HCHXZZ, 0HCJXZZ, 0HRDXK3, 0HRDXK4, 0NU607Z, 0NU60JZ, 0QTB0ZZ, 0RNNXZZ, 0RPD0JZ, 0SGF37Z, 0SS7X4Z, 0UCB7ZZ, 2W09X2Z, 3E0F7KZ, 4A033BF, 5A09559, 5A0955B, BR2C1ZZ, BT011ZZ, BT01YZZ |
| Colectomy | ICD-9 | 17.3, 17.31, 17.32, 17.33, 17.34, 17.35, 17.36, 17.39, 45.41, 45.71, 45.72, 45.73, 45.74, 45.75, 45.76, 45.79, 45.7, 48.61, 45.8, 45.81, 45.82, 45.83 |
|  | ICD-10 | 0DBC0ZX, 0DBC0ZZ, 0DBC3ZX, 0DBC3ZZ, 0DBC4ZX, 0DBC4ZZ, 0DBC7ZX, 0DBC7ZZ, 0DBE0ZX, 0DBE0ZZ, 0DBE3ZX, 0DBE3ZZ, 0DBE4ZX, 0DBE4ZZ, 0DBE7ZX, 0DBE7ZZ, 0DBF0ZX, 0DBF0ZZ, 0DBF3ZX, 0DBF3ZZ, 0DBF4ZX, 0DBF4ZZ, 0DBF7ZX, 0DBF7ZZ, 0DBG0ZX, 0DBG0ZZ, 0DBG3ZX, 0DBG3ZZ, 0DBG4ZX, 0DBG4ZZ, 0DBG7ZX, 0DBG7ZZ, 0DBGFZZ, 0DBH0ZX, 0DBH0ZZ, 0DBH3ZX, 0DBH3ZZ, 0DBH4ZX, 0DBH4ZZ, 0DBH7ZX, 0DBH7ZZ, 0DBK0ZX, 0DBK0ZZ, 0DBK3ZX, 0DBK3ZZ, 0DBK4ZX, 0DBK4ZZ, 0DBK7ZX, 0DBK7ZZ, 0DBL0ZX, 0DBL0ZZ, 0DBL3ZX, 0DBL3ZZ, 0DBL4ZX, 0DBL4ZZ, 0DBL7ZX, 0DBL7ZZ, 0DBLFZZ, 0DBM0ZX, 0DBM0ZZ, 0DBM3ZX, 0DBM3ZZ, 0DBM4ZX, 0DBM4ZZ, 0DBM7ZX, 0DBM7ZZ, 0DBMFZZ, 0DBN0ZX, 0DBN0ZZ, 0DBN3ZX, 0DBN3ZZ, 0DBN4ZX, 0DBN4ZZ, 0DBN7ZX, 0DBN7ZZ, 0DBNFZZ, 0DTC0ZZ, 0DTC4ZZ, 0DTC7ZZ, 0DTC8ZZ, 0DTF0ZZ, 0DTF4ZZ, 0DTF7ZZ, 0DTF8ZZ, 0DTG0ZZ, 0DTG4ZZ, 0DTG7ZZ, 0DTG8ZZ, 0DTGFZZ, 0DTH0ZZ, 0DTH4ZZ, 0DTH7ZZ, 0DTH8ZZ, 0DTK0ZZ, 0DTK4ZZ, 0DTK7ZZ, 0DTK8ZZ, 0DTL0ZZ, 0DTL4ZZ, 0DTL7ZZ, 0DTL8ZZ, 0DTLFZZ, 0DTM0ZZ, 0DTM4ZZ, 0DTM7ZZ, 0DTM8ZZ, 0DTMFZZ, 0DTN0ZZ, 0DTN4ZZ, 0DTN7ZZ, 0DTN8ZZ, 0DTNFZZ, 0DTE0ZZ, 0DTE4ZZ, 0DTE7ZZ, 0DTE8ZZ |
| Colonoscopy | ICD-9 | 45.23, 98.04 |
|  | ICD-10 | 0DBC8ZX, 0DBC8ZZ, 0DBF8ZX, 0DBF8ZZ, 0DBH8ZX, 0DBH8ZZ, 0DBK8ZX, 0DBK8ZZ, 0DBL8ZX, 0DBL8ZZ, 0DBM8ZX, 0DBM8ZZ |
| CT Colonography | ICD-10 | BD2400Z, BD240ZZ, BD2410Z, BD241ZZ, BD24Y0Z, BD24YZZ, BD24ZZZ |
| Lower endoscopy NOS | ICD-9 | 45.22, 45.25, 45.42, 45.43, 48.24, 48.36 |
|  | ICD-10 | 0DBE8ZX, 0DBE8ZZ, 0DBG8ZX, 0DBG8ZZ, 0DBN8ZX, 0DBN8ZZ, 0DBP8ZX, 0DBP8ZZ, 0DJD8ZZ |
| Proctectomy | ICD-9 | 48.35, 48.4, 48.41, 48.42, 48.43, 48.49, 48.4, 48.5, 48.5, 48.51, 48.52, 48.59, 48.6, 48.62, 48.63, 48.64, 48.65, 48.69 |
|  | ICD-10 | 0DBP0ZX, 0DBP0ZZ, 0DBP3ZX, 0DBP3ZZ, 0DBP4ZX, 0DBP4ZZ, 0DBP7ZX, 0DBP7ZZ, 0DTP0ZZ, 0DTP4ZZ, 0DTP7ZZ, 0DTP8ZZ |
| Sigmoidoscopy | ICD-9 | 45.24, 48.21, 48.22, 48.23 |

Figure 1: Cohort Assembly


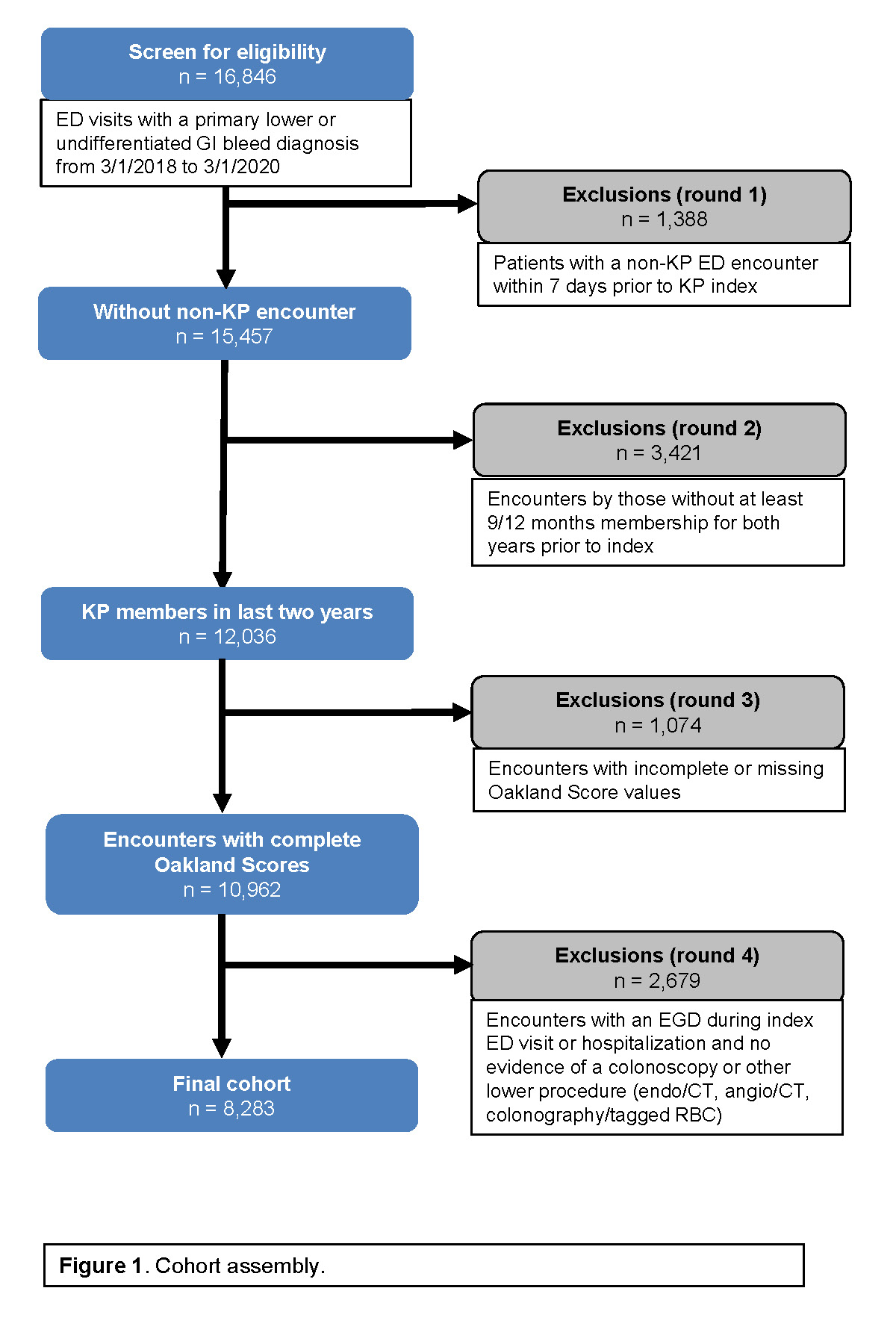


Figure 2: Variable Importance: Logistic Regression Model


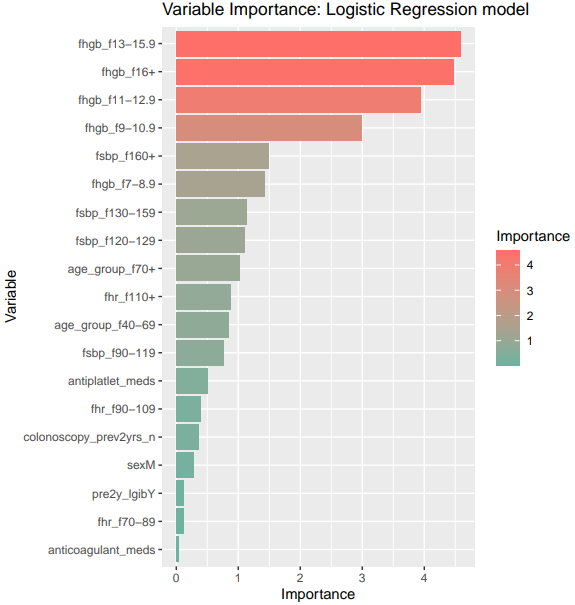


Notes: Abbreviations: fhgb; first hemoglobin; fsbp; first blood pressure; fhr; first heart rate; prev2yrs, previous two years
